# Situationally Selective Activation of Moral Disengagement Mechanisms in School Bullying: A Repeated Within-Subjects Experimental Study

**DOI:** 10.3389/fpsyg.2020.01101

**Published:** 2020-06-09

**Authors:** Robert Thornberg, Elina Daremark, Jonn Gottfridsson, Gianluca Gini

**Affiliations:** ^1^Department of Behavioural Sciences and Learning, Linköping University, Linköping, Sweden; ^2^Department of Development and Social Psychology, University of Padua, Padua, Italy

**Keywords:** moral disengagement, bullying, moral justification, euphemistic labeling, displacement of responsibility, diffusion of responsibility, victim blaming, dehumanization

## Abstract

With reference to social–cognitive theory, the aim of the present study was to examine whether school students’ tendency to display different moral disengagement mechanisms varies according to different social cues in hypothetical events in which they are engaged in bullying behavior. A repeated within-subjects experimental design was adopted. A total of 706 Swedish students (aged 10–20) from 75 classrooms responded to four verbal bullying vignettes by filling out a self-report survey. The results showed changes in moral disengagement mechanisms across the bullying situations. For instance, moral justification, victim blaming, and dehumanization scored higher in the mean victim condition and lower in the likable victim condition than in the other two conditions. Diffusion of responsibility was higher in the group conformity condition than in the other conditions. The findings also revealed differences in the levels of moral disengagement mechanisms within the bullying conditions. For example, euphemistic labeling and displacement of responsibility scored higher than the other mechanisms in the laughing audience condition. Victim blaming scored higher than the other mechanisms in the mean victim condition. Dehumanization, victim blaming, and moral justification scored lowest while euphemistic labeling was higher than most of the other mechanisms in the likable victim condition.

## Introduction

Bullying is usually defined as repeated aggressive, offensive, or inhumane behavior directed at individuals who are disadvantaged or less powerful in relation to the perpetrator(s) (Olweus, 1993; Jimerson et al., 2010) and can therefore be categorized as “unfair and immoral behavior” (Romera et al., 2019). Accordingly, research has found that children judge bullying to be a serious transgression and wrong independently of school rules by referring to the harm it causes the victim (Thornberg, 2010; Thornberg et al., 2016, 2017). Still, it is a worldwide problem in schools (Chester et al., 2015). Thus, there is a gap between moral standards and actions, but Bandura (2016) argues that the theoretical understanding of moral agency cannot be reduced to moral standards but has to include motivational and self-regulatory processes that could either translate moral standards into moral action or create barriers between standards and action. These self-regulatory processes are *situated* cognitive processes (Bandura, 2016) that may be influenced by specific features of the social interaction that takes place. Therefore, the aim of the current study was to examine whether adolescents’ tendency to display different moral disengagement mechanisms varies across different (hypothetical) social situations, where characteristics of the situations were experimentally manipulated.

### Social–Cognitive Theory of Moral Agency

According to the social–cognitive theory of moral agency (Bandura, 1999, 2016), moral standards are linked to moral actions through self-regulation processes that monitor, regulate, and evaluate individuals’ actions. These self-regulation processes produce self-sanctions (i.e., a sense of guilt, remorse, and self-condemnation) when individuals recognize themselves as doing wrong, and self-approval (i.e., a sense of self-worth) when they recognize themselves as doing right. The self-regulation of morality cannot, however, be reduced to intrapsychic processes but is embedded in a dynamic social context. “Moral agency is socially situated and exercised in particularized ways” (Bandura, 1999, p. 207). The social–cognitive theory adopts an interactionist perspective on morality, in which situational and contextual factors are important.

To understand the gap between moral standards and actions, Bandura (1999, 2016) proposes the concept of *moral disengagement*, which refers to a set of self-serving cognitive distortions by which self-regulating mechanisms can be deactivated and moral self-sanctions can be disengaged, which in turn makes it possible to behave inhumanely or aggressively without feelings of guilt or remorse. Individuals justify immoral conduct in order to help themselves avoid the self-sanctions that would typically follow from such conduct. In this way, moral disengagement facilitates and promotes inhumane behaviors. Previous research has shown that children and adolescents who bully others more than their peers do tend to display greater moral disengagement than their peers (e.g., Gini, 2006; Gini et al., 2011; Caravita et al., 2012; Bussey et al., 2015; Pozzoli et al., 2016; for meta-analyses, see Gini et al., 2014; Killer et al., 2019).

Specifically, moral disengagement includes eight mechanisms that could be selectively activated in a given situation: (a) *moral justification:* using worthy ends or moral purposes to sanction pernicious means, (b) *euphemistic labeling:* labeling the inhumane or aggressive behavior in a way that makes the act sound less negative or more respectable, (c) *advantageous comparison:* making a bad act seem less bad and more acceptable by comparing it to a worse act, (d) *displacement of responsibility:* minimizing personal responsibility by viewing one’s actions as stemming from authorities, (e) *diffusion of responsibility:* diluting personal responsibility due to the presence or involvement of other people, (f) *distorting the consequences:* perceptually minimizing, ignoring, or misconstruing the negative or harmful effects of the inhumane behavior, (g) *dehumanization:* stripping the victim of human qualities and equal value, and (h) *victim blaming:* viewing the victim as responsible for his or her own suffering (Bandura, 1999, 2016).

While moral disengagement is theoretically and conceptually a multidimensional construct, as described above, the vast majority of studies in the field of aggression and bullying have examined and measured it as a unidimensional construct (Gini et al., 2014; Killer et al., 2019). Therefore, there is still not much knowledge about the degree to which different moral disengagement mechanisms may be related to bullying perpetration. Even though all or most of the foci or mechanisms of moral disengagement might positively correlate with bullying perpetration (e.g., Thornberg and Jungert, 2014; Zych et al., 2019), only a few studies have examined their associations with bullying perpetration when all mechanisms are included in the same models. In their regression models, Robson and Witenberg (2013) found that moral justification and diffusion of responsibility were significantly associated with traditional bullying behavior, whereas diffusion of responsibility and blaming the victim were significantly associated with cyberbullying behavior. Thornberg and Jungert (2014) found that bullying perpetration was linked to victim attribution (dehumanization and victim blaming merged into one factor after factor analysis) and moral justification in a sample of Swedish pre-adolescent students. These findings were then replicated in a recent study of direct bullying perpetration, while indirect bullying perpetration was only related to victim attribution (Bjärehed et al., 2020). In their study, Oliveira et al. (2019) found that bullying behavior was associated with victim blaming, dehumanization, and displacement of responsibility.

### Situated Selective Activation of Moral Disengagement Mechanisms

Social–cognitive theory posits that moral disengagement is a product of the reciprocal interplay between individual and social influences (Bandura, 1999, 2016). It should therefore be considered as consisting of *situated* cognitive processes. “Moral disengagement is not a dispositional trait that can be assessed by a one-size-fits-all measure. Disengagement mechanisms operate across different aspects of life, but they are manifested differently depending on the sphere of activity” (Bandura, 2016, p. 26). Bandura (1999, 2016) argues that moral disengagement mechanisms can be selectively activated under different circumstances. However, to our knowledge, previous research on moral disengagement and bullying perpetration has examined and measured moral disengagement by considering individuals’ (or groups’) propensities to morally disengage as more or less stable attitudes or trait-like cognitive distortions and by using a general attitude-like scale (e.g., Hymel et al., 2005; Robson and Witenberg, 2013; Cuadrado-Gordillo and Fernández-Antelo, 2019; Oliveira et al., 2019; Thornberg et al., 2019).

In their recent meta-analysis of moral disengagement in bullying perpetration, Killer et al. (2019) conclude that there is a lack of examining the wider influence of the situational context. Therefore, they suggest as a future direction: “While MD and contextual variables have been known to interact to impact aggressive behavior, further research into this interaction specifically in bullying scenarios would further enhance our understanding of the role MD plays in bullying-related behaviors” (Killer et al., 2019, p. 458). We would like to add that it would be plausible, in particular, to assume that different mechanisms of moral disengagement could be activated to a greater or lesser extent due to variations in the characteristics or “cues” across bullying situations.

#### The Mean Victim

Moral justification is a powerful mechanism through which actors interpret their aggressive behavior as serving a moral purpose, which influences them to consider themselves moral agents who can feel good about themselves (i.e., self-approval) while inflicting harm on others (Bandura, 2016). Bandura (1999) argues that moral justification is most strikingly revealed in military conduct in which people “see themselves as fighting ruthless oppressors, protecting their cherished values, preserving world peace, saving humanity from subjugation, or honoring their country’s commitment” (p. 195). In a school bullying context, if bullies interpret the victim as someone who has been mean to others, in particular to their friends, such a “cue” could easily trigger or activate moral justification in which they see themselves as standing up for and protecting their friends by fighting the victim. Furthermore, this *mean victim* condition might trigger or activate victim blaming even more—the bullies perceive that the victims deserve their suffering and are at fault for bringing maltreatment on themselves. In addition, it might be easier to dehumanize a mean or aggressive victim by reducing them to a worthless “jerk,” “douchebag,” “idiot,” or “savage.” Through this dehumanization process, bullies become less inclined to identify or empathize with the victim and more motivated and prone to act inhumanely and aggressively toward him or her (Haslam and Loughnan, 2014; Li et al., 2014; Webster and Saucier, 2015; Bandura, 2016).

#### Group Conformity

In contrast, it is plausible to assume that moral disengagement mechanisms that minimize or obscure each peer-group member’s personal responsibility and agency in causing harm would be more easily activated if a peer group, instead of a single perpetrator, is bullying a victim. Perhaps most obviously, bullying performed by a group of students can trigger diffusion of responsibility (Olweus, 1993; Bandura, 1999, 2016) as “any harm done by the group can always be attributed largely to the behaviors of others” (Bandura, 1999, p. 198). Moreover, if it is perceived as possible for a whole peer group to bully someone at school without teacher intervention, this condition could activate displacement of responsibility in terms of viewing the school authorities as being responsible for what is going on (e.g., “It’s the teachers’ fault because they aren’t there and stopping it.”).

If teachers and playground supervisors fail to detect and promptly intervene in bullying, and if there are no effective consequences for the bullies, students will probably define this as a sign of poor and ineffective anti-bullying practice conducted by the authorities in school (Cunningham et al., 2016) that in fact makes room for bullying. According to a recent qualitative study examining how students understand and explain bullying (Thornberg and Delby, 2019), if they perceive that teachers and other school staff allow bullying to take place, then the lesson learned is that bullying is an accepted behavior at school and is not banned by school rules. Bullying conducted by a single student would probably be more easily dismissed as a deviant student’s work and an exception to the rule, whereas displacement of responsibility onto the teachers and other school staff is more likely to occur when bullying is conducted by whole peer groups within the school.

#### Laughing Audience

Another bullying condition that might increase the likelihood of activating displacement of responsibility is when there are peers present who are laughing and cheering the bully on. By taking the participant role of reinforcer (Salmivalli, 2010), these bystanders socially approve and reward the bully. They are telling him or her: “We like this! You’re doing the right thing!” As in the situation in which a whole peer group is engaged in bullying, the bully who is repeatedly supported and cheered on by peers will be more inclined to interpret the bullying perpetration as socially accepted and allowed in school and that therefore it is the teachers and other school staff who are responsible because they allow it to happen (otherwise, they should have noticed, intervened, and stopped the bullying).

In addition, bystanders who are laughing and cheering will probably trigger or activate euphemistic labeling, interpreting bullying as a non-serious joke or playful act. In a qualitative study (Thornberg and Delby, 2019), some students themselves recognized that bystanders can contribute to “normalizing” bullying by co-constructing it as “joking” when they smile and laugh. They help the bully to blur the boundary between joking and abuse and to view the bullying as just an expression of a trivial, normal, and ordinary joking jargon. When all the peers around are smiling and laughing, it will be easier for the bully to think, “I’m just joking,” and “this is just for fun.” When a group of students are laughing and smiling, it might even influence any teacher present to misinterpret what they are doing as humor, joking, and having fun instead of bullying and therefore do nothing to intervene (Smith-Adcock et al., 2019; Thornberg and Delby, 2019), which further facilitates displacement of responsibility among the students.

#### The Likable Victim

If students are bullying someone whom they actually like and perceive as kind to everyone, they are likely to be less inclined to dehumanize and blame the victim than in the three bullying conditions above. The victim cannot simply be stripped of his or her human qualities and equal value, reduced to a negative label, or blamed but is perceived as deserving to be treated well and is at a lower risk of victimization (Babarro et al., 2017; Wang et al., 2019; Ma et al., 2020). In fact, bullying someone who is liked, friendly, and kind to others is so obviously wrong and unjustifiable that, for most children and adolescents, engaging in such behavior would inevitably create cognitive dissonance, resulting in aversive arousal (Festinger, 1962; McGrath, 2017), feelings of guilt and self-condemnation (Bandura, 1999, 2016), and moral distress (cf., Brüggemann et al., 2019; Gini et al., 2020). It should in fact be more difficult for moral disengagement in general to be activated to convince the actor that the inhumane behavior is acceptable because of the actor’s friendly feelings for the victim and perception of the victim as a good person. In addition to dehumanization and victim blaming, mechanisms like moral justification, advantageous comparison, and diffusion of responsibility appear to be rather unconvincing. However, one way out might be to talk about and reinterpret the whole situation and one’s behavior as something other than mean behavior in general, and bullying in particular. The perpetrator can use labels like “friendly teasing” and “just joking” to make “humor come up smelling fresh and friendly” (Billig, 2005, p. 25). Therefore, we assume that students would be more inclined to use euphemistic labeling than other moral disengagement mechanisms in the likable victim condition because it helps them to reduce cognitive dissonance and the associated unwanted negative emotions by convincing them that they are not being mean and aggressive toward the well-liked peer but are “just joking.”

### Aim and Hypotheses

In accordance with the social–cognitive theory (Bandura, 1999, 2016), the aim of the present study was to examine whether school students’ tendency to display different moral disengagement mechanisms varies due to different social cues in hypothetical events in which they are engaged in bullying behavior. Although we deduced a set of hypotheses based on the literature review, the current study is, as far as we know, the first to use an experimental design to examine the degree to which various moral disengagement mechanisms might be situated and thus affected by various social cues in bullying situations. The current study should therefore be considered rather exploratory, and its hypotheses weak.

We proposed the following hypotheses about the tendencies of moral disengagement mechanisms across bullying conditions: In hypothetical bullying situations in which students are acting as bullies: (1) students score higher in *moral justification* in the mean victim condition than in the other conditions; (2) students score higher in *euphemistic labeling* in the laughing audience and likable victim conditions than in the other conditions; (3) students score higher in *displacement of responsibility* in the group conformity and laughing audience conditions than in the other conditions; (4) students score higher in *diffusion of responsibility* in the group conformity condition than in the other conditions; (5) students score higher in *dehumanization* in the mean victim condition than in the other conditions and lower in the likable victim condition than in the other conditions; and (6) students score higher in *victim blaming* in the mean victim condition than in the other conditions and lower in the likable victim condition than in the other conditions. How *advantageous comparison* and *distorting consequences* may vary across the hypothetical bullying conditions was examined in an exploratory manner.

Furthermore, we proposed the following hypotheses about the tendencies of moral disengagement mechanisms within bullying conditions: In hypothetical bullying situations in which students are acting as bullies: (1) in the *group conformity* condition, students score higher in displacement of responsibility and diffusion of responsibility than in other mechanisms; (2) in the *laughing audience* condition, students score higher in displacement of responsibility and euphemistic labeling than in other mechanisms; (3) in the *mean victim* condition, students score higher in moral justification and victim blaming than in other mechanisms; and (4) in the *likable victim* condition, students score higher in euphemistic labeling and lower in dehumanization and victim blaming than in other mechanisms.

## Materials and Methods

In the current study, we adopted a repeated within-subjects experimental design using a vignette technique in which the same participants took part in each condition of the experiment.

### Participants

Participants were recruited from 20 schools in Sweden. A non-probability two-step sampling was used in the study. First, a purposive sampling of schools was carried out, which led to the inclusion of 20 schools representing various socio-geographic and socioeconomic positions, and including upper elementary schools (students are usually around 10–13 years old) and secondary schools (students are usually around 13–19 years old). In the next step, we conducted a convenience sampling of students in these grades and years. The original sample consisted of 1,695 students [911 (54%) boys and 784 (46%) girls]. Parental consent letters were distributed to all the families (parental consent reached 43%). All the participants were asked for their own consent in addition to parental consent. Six students did not participate because they did not want to, and nine students were excluded from the study because they did not complete the questionnaire.

Thus, the final sample consisted of 706 students located in 75 classrooms in 20 schools, resulting in a participation rate of 42%. The sampling data indicate selection bias due to gender differences between the original and final sample. In the final sample, 310 (44%) students reported a male gender, 386 (55%) students reported a female gender, and 10 (1%) students reported “other” gender (note that gender data are based on biological sex in the original sample, and on self-reports in the final sample). The age range in the final sample was 10–20 years old (*M* = 14.5, *SD* = 2.85) with 326 students in upper elementary school and 380 in secondary school. The two-step sampling procedure led to a sample of students from different socioeconomic (ranging from lower to upper-middle class) and socio-geographic backgrounds. Most of the participants were of Swedish ethnicity, while a minority (6%) had a foreign background; in other words, they were born in another country and/or both parents were born in another country. We obtained active parental consent and student consent for all 706 participants.

### Procedures and Measures

Data were collected from a web-based, anonymous, self-report questionnaire, which the participants filled in on a tablet, computer, or cellphone in their regular classrooms. Either the second or the third author was present throughout the session to explain the study procedure and assist the participants. They are both trained graduate students in psychology. To ensure anonymity, the participants were instructed to move away from each other and to separate their desks. The procedure took about 30–40 min in each classroom. The study received ethical approval from the Regional Ethical Review Board at Linköping.

#### Bullying Vignettes

The questionnaire consisted of four bullying vignettes (hypothetical scenarios) that did not mention the word “bullying” (see Appendix)^1^. Before the four vignettes, it was stated in the questionnaire under the heading, “Pretend that you are a person who is teasing others”: “Here come four short stories that we want you to identify yourself with. We want you to pretend to be a person who teases others and who is more popular, powerful and stronger than the person you are teasing. Don’t worry about whether you have done any of these things before or not. Just imagine in each story that it is you who is doing it.” Because a repeated within-subject experimental design is vulnerable to order effects and carryover effects, two versions of the questionnaires (A and B) were constructed.

In questionnaire A, the bullying vignettes were presented in the following order: (1) group conformity, (2) laughing audience, (3) mean victim, and (4) likable victim (see Appendix). In questionnaire B, the order was the opposite. Within each classroom, approximately half of the participants were randomly assigned to fill out questionnaire A and the other half to fill out questionnaire B. The victim and other included people in the vignettes were gender-neutral: their sex was not mentioned.

#### Moral Disengagement

The participants were asked to fill out a 16-item moral disengagement scale after each vignette (see Appendix). The scale was developed for the present study and consisted of two items for each mechanism. In order to cope with order effect and carryover effect, the scale had an A-B-C-D-E-F-G-H-H-G-F-E-D-C-B-A design (A = euphemistic labeling, B = diffusion of responsibility, C = victim blaming, D = distorting the consequences, E = dehumanization, F = advantageous comparison, G = displacement of responsibility, H = moral justification). Participants rated each item on a seven-point scale (1 = strongly disagree to 7 = strongly agree). The Spearman–Brown coefficient was 0.83, 0.83, 0.80, and 0.90 for euphemistic labeling, 0.80, 0.84, 0.84, and 0.82 for diffusion of responsibility, 0.82, 0.90, 0.88, and 0.81 for victim blaming, 0.78, 0.80, 0.79, and 0.82 for distorting the consequences, 0.74, 0.81, 0.74, and 0.76 for dehumanization, 0.86, 0.87, 0.91, and 0.87 for advantageous comparison, 0.87, 0.86, 0.89, and 0.90 for displacement of responsibility, and 0.82, 0.83, 0.87, and 0.88 for moral justification. Cronbach’s α reliability for the moral disengagement scale in each vignette was 0.94, 0.95, 0.94, and 0.94.

## Results

Descriptive statistics for the eight moral disengagement mechanisms in each of the four bullying vignettes are presented in Table 1. No hypotheses about gender or age differences were made in the present study; therefore, gender and age were not included in the analyses. One-way ANOVAs with repeated measures were computed to test the hypotheses. Mauchly’s test indicated that the assumption of sphericity was violated in all ANOVAs. Therefore, degrees of freedom were corrected using Greenhouse–Geisser estimate when estimates of sphericity were less than 0.75, whereas Huynh-Feldt estimate was used when estimates were greater than 0.75 (Field, 2013). Cohen’s *d*_*z*_ (calculated from the *t*-value using a dependent *t*-test and *N*; see Lakens, 2013) was calculated as effect size in the *post hoc* tests.

**TABLE 1:** Means (*M*), Standard Deviations (*SD*), and Mean Differences (*F* and partial η^2^) for moral disengagement across the bullying situations.

**Mechanisms**	**Group conformity**	**Laughing audience**	**Mean victim**	**Likable victim**	***F***	**partial η^2^**
Moral disengagement	1.88 (1.08)	1.92 (1.12)	2.36 (1.26)	1.75 (1.01)	182.73^b^	0.21

*^*b*^Huynh-Feldt estimate of sphericity; the ANOVA reached statistical significance (*p* < 0.001).*

Before testing our hypotheses, one-way, repeated-measure ANOVAs were performed to examine whether moral disengagement as a global construct varies across the four bullying conditions. The results showed a significant main effect (Table 1). Bonferroni *post hoc* procedures revealed that moral disengagement was higher in the mean victim condition than in the likable victim condition (*p* < 0.001, Cohen’s *d_*z*_* = 0.74), the group conformity condition (*p* < 0.001, Cohen’s *d_*z*_* = 0.72), and the laughing audience condition (*p* < 0.001, Cohen’s *d_*z*_* = 0.70). In addition, moral disengagement scored lower in the likable victim condition than in the laughing audience condition (*p* < 0.001, Cohen’s *d_*z*_* = −0.15) and the group conformity condition (*p* < 0.001, Cohen’s *d_*z*_* = −0.08).

### Changes in Moral Disengagement Mechanisms Across the Bullying Situations

In order to test our hypotheses about the tendencies of moral disengagement mechanisms across bullying conditions, changes in each of the eight mechanisms for moral disengagement across the four bullying situations were analyzed. Eight one-way repeated-measure ANOVAs were performed—one for each mechanism—using the mean values for each of the four bullying conditions (presented horizontally in Table 2).

**TABLE 2:** Means (*M*) and Standard Deviations (*SD*) for moral disengagement mechanisms and mean differences (*F* and partial η^2^) across and within the bullying situations.

**Mechanisms**	**Group conformity**	**Laughing audience**	**Mean victim**	**Likable victim**	***F***	**partial η^2^**
Moral justification	1.53 (1.08)	1.61 (1.13)	2.72 (1.78)	1.45 (1.05)	271.57^a^	0.28
Euphemistic labeling	2.08 (1.53)	2.31 (1.66)	2.09 (1.48)	2.24 (1.76)	10.74^b^	0.02
Advantageous comparison	2.08 (1.46)	2.10 (1.53)	2.50 (1.81)	1.98 (1.51)	54.60^b^	0.07
Displacement of responsibility	2.34 (1.60)	2.38 (1.63)	2.30 (1.61)	2.10 (1.53)	33.61^b^	0.02
Diffusion of responsibility	2.14 (1.54)	1.97 (1.48)	1.97 (1.48)	1.65 (1.25)	47.08^b^	0.06
Distorting consequences	1.86 (1.30)	1.92 (1.37)	2.22 (1.53)	1.94 (1.46)	26.42^b^	0.04
Dehumanization	1.46 (1.03)	1.48 (1.06)	1.64 (1.19)	1.30 (0.84)	32.57^b^	0.04
Victim blame	1.53 (1.10)	1.56 (1.18)	3.47 (2.07)	1.38 (0.98)	582.01^a^	0.45
*F*	104.98^a^	104.22^a^	171.59^a^	104.17^a^		
partial η^2^	0.13	0.13	0.20	0.13		

*The eight ANOVAs testing mean differences across the bullying situations are presented horizontally; the four ANOVAs testing mean differences within the bullying situations are presented vertically; ^*a*^Greenhouse–Geisser estimate of sphericity; ^*b*^Huynh–Feldt estimate of sphericity; all ANOVAs reached statistical significance (*p* < 0.001).*

#### Moral Justification

The first ANOVA showed a significant main effect. Bonferroni *post hoc* procedures revealed that moral justification was higher in the mean victim condition than in the likable victim condition (*p* < 0.001, Cohen’s *d_*z*_* = 0.74), the group conformity condition (*p* < 0.001, Cohen’s *d_*z*_* = 0.72), and the laughing audience condition (*p* < 0.001, Cohen’s *d_*z*_* = 0.70). In addition, moral justification scored higher in the laughing audience condition than in the likable victim condition (*p* = 0.001, Cohen’s *d_*z*_* = 0.15).

#### Euphemistic Labeling

The second ANOVA indicated a significant main effect. Bonferroni *post hoc* procedures revealed that euphemistic labeling was higher in the laughing audience condition and the likable victim condition than in the group conformity condition (*p* < 0.001, Cohen’s *d_*z*_* = 0.21; *p* = 0.019, Cohen’s *d_*z*_* = 0.11) and the mean victim condition (*p* < 0.001, Cohen’s *d_*z*_* = 0.19; *p* = 0.043, Cohen’s *d_*z*_* = 0.10).

#### Advantageous Comparison

The third ANOVA demonstrated a significant main effect. Bonferroni *post hoc* procedures revealed that advantageous comparison was significantly higher in the mean victim condition than in the likable victim condition (*p* < 0.001, Cohen’s *d_*z*_* = 0.39), the laughing audience condition (*p* < 0.001, Cohen’s *d_*z*_* = 0.36), and the group conformity condition (*p* < 0.001, Cohen’s *d_*z*_* = 0.33). In addition, advantageous comparison scored higher in the laughing audience condition than in the likable victim condition (*p* = 0.020, Cohen’s *d_*z*_* = 0.11).

#### Displacement of Responsibility

The fourth ANOVA showed a significant main effect. Bonferroni *post hoc* procedures revealed that displacement of responsibility was lower in the likable victim condition than in the laughing audience condition (*p* < 0.001, Cohen’s *d_*z*_* = −0.24), the group conformity condition (*p* < 0.001, Cohen’s *d_*z*_* = −0.20), and the mean victim condition (*p* < 0.001, Cohen’s *d_*z*_* = −0.19).

#### Diffusion of Responsibility

The fifth ANOVA showed a significant main effect. Bonferroni *post hoc* procedures revealed that diffusion of responsibility was higher in the group conformity condition than in the likable victim condition (*p* < 0.001, Cohen’s *d_*z*_* = 0.38), the laughing audience condition (*p* < 0.001, Cohen’s *d_*z*_* = 0.16), and the mean victim condition (*p* = 0.001, Cohen’s *d_*z*_* = 0.14). Furthermore, diffusion of responsibility scored lower in the likable victim condition than in the mean victim condition (*p* < 0.001, Cohen’s *d_*z*_* = −0.32) and the laughing audience condition (*p* < 0.001, Cohen’s *d_*z*_* = −0.28).

#### Distorting Consequences

The sixth ANOVA demonstrated a significant main effect. Bonferroni *post hoc* tests using the correction revealed that distorting consequences were significantly higher in the mean victim condition than in the group conformity condition (*p* < 0.001, Cohen’s *d_*z*_* = 0.32), the laughing audience condition (*p* < 0.001, Cohen’s *d_*z*_* = 0.27), and the likable victim condition (*p* < 0.001, Cohen’s *d_*z*_* = 0.21).

#### Dehumanization

The seventh ANOVA showed a significant main effect. Bonferroni *post hoc* procedures revealed that dehumanization scored higher in the mean victim condition than in the likable victim condition (*p* < 0.001, Cohen’s *d_*z*_* = 0.31), the group conformity condition (*p* < 0.001, Cohen’s *d_*z*_* = 0.19), and the laughing audience condition (*p* < 0.001, Cohen’s *d_*z*_* = 0.18). In addition, dehumanization was significantly lower in the likable victim condition than in the laughing audience condition (*p* < 0.001, Cohen’s *d_*z*_* = −0.21) and the group conformity condition (*p* < 0.001, Cohen’s *d_*z*_* = −0.17).

#### Victim Blaming

The eighth ANOVA indicated a significant main effect. Bonferroni *post hoc* procedures revealed that victim blaming was higher in the mean victim condition than in the likable victim condition (*p* < 0.001, Cohen’s *d_*z*_* = 1.02), the group conformity condition (*p* < 0.001, Cohen’s *d_*z*_* = 0.99), and the laughing audience condition (*p* < 0.001, Cohen’s *d_*z*_* = 0.97). In addition, victim blaming scored lower in the likable victim condition than in the laughing audience condition (*p* < 0.001, Cohen’s *d_*z*_* = −0.18) and the group conformity condition (*p* = 0.001, Cohen’s *d_*z*_* = −0.15).

### Differences in Moral Disengagement Mechanisms Within the Bullying Situations

In order to test the hypotheses about the tendencies of moral disengagement mechanisms within bullying conditions, mean differences between the eight mechanisms for moral disengagement in each of the four bullying situations were analyzed. Four one-way repeated-measure ANOVAs were performed—one for each bullying situation—using the mean values for each of the eight mechanisms (presented vertically in Table 2).

#### Group Conformity

The first ANOVA showed a significant main effect. Bonferroni *post hoc* procedures revealed that displacement of responsibility was higher than dehumanization (*p* < 0.001, Cohen’s *d_*z*_* = 0.60), victim blaming (*p* < 0.001, Cohen’s *d_*z*_* = 0.54), moral justification (*p* < 0.001, Cohen’s *d_*z*_* = 0.54), distorting consequences (*p* < 0.001, Cohen’s *d_*z*_* = 0.31), advantageous comparison (*p* < 0.001, Cohen’s *d_*z*_* = 0.17), euphemistic labeling (*p* = 0.003, Cohen’s *d_*z*_* = 0.15), and diffusion of responsibility (*p* = 0.026, Cohen’s *d_*z*_* = 0.12).

Furthermore, diffusion of responsibility, euphemistic labeling, and advantageous comparison scored higher than dehumanization (*p* < 0.001, Cohen’s *d_*z*_* = 0.53; *p* < 0.001, Cohen’s *d_*z*_* = 0.53; *p* < 0.001, Cohen’s *d_*z*_* = 0.54), victim blaming (*p* < 0.001, Cohen’s *d_*z*_* = 0.49; *p* < 0.001, Cohen’s *d_*z*_* = 0.51; *p* < 0.001, Cohen’s *d_*z*_* = 0.50), moral justification (*p* < 0.001, Cohen’s *d_*z*_* = 0.45; *p* < 0.001, Cohen’s *d_*z*_* = 0.46; *p* < 0.001, Cohen’s *d_*z*_* = 0.47), and distorting consequences (*p* < 0.001, Cohen’s *d_*z*_* = 0.23; *p* < 0.001, Cohen’s *d_*z*_* = 0.25; *p* < 0.001, Cohen’s *d_*z*_* = 0.24). Finally, distorting consequences were higher than dehumanization (*p* < 0.001, Cohen’s *d_*z*_* = 0.39), victim blaming (*p* < 0.001, Cohen’s *d_*z*_* = 0.34), and moral justification (*p* < 0.001, Cohen’s *d_*z*_* = 0.33).

#### Laughing Audience

The second ANOVA showed a significant main effect. Bonferroni *post hoc* procedures revealed that euphemistic labeling and displacement of responsibility were higher than dehumanization (*p* < 0.001, Cohen’s *d_*z*_* = 0.61; *p* < 0.001, Cohen’s *d_*z*_* = 0.59), victim blaming (*p* < 0.001, Cohen’s *d_*z*_* = 0.57; *p* < 0.001, Cohen’s *d_*z*_* = 0.53), moral justification (*p* < 0.001, Cohen’s *d_*z*_* = 0.55; *p* < 0.001, Cohen’s *d_*z*_* = 0.50), distorting consequences (*p* < 0.001, Cohen’s *d_*z*_* = 0.39; *p* < 0.001, Cohen’s *d_*z*_* = 0.29), diffusion of responsibility (*p* < 0.001, Cohen’s *d_*z*_* = 0.26; *p* < 0.001, Cohen’s *d_*z*_* = 0.26), and advantageous comparison (*p* < 0.001, Cohen’s *d_*z*_* = 0.18; *p* < 0.001, Cohen’s *d_*z*_* = 0.17).

Moreover, advantageous comparison scored higher than dehumanization (*p* < 0.001, Cohen’s *d_*z*_* = 0.51), victim blaming (*p* < 0.001, Cohen’s *d_*z*_* = 0.47), moral justification (*p* < 0.001, Cohen’s *d_*z*_* = 0.43), and distorting consequences (*p* < 0.001, Cohen’s *d_*z*_* = 0.19). Diffusion of responsibility and distorting consequences were higher than dehumanization (*p* < 0.001, Cohen’s *d_*z*_* = 0.42; *p* < 0.001, Cohen’s *d_*z*_* = 0.40), victim blaming (*p* < 0.001, Cohen’s *d_*z*_* = 0.37; *p* < 0.001, Cohen’s *d_*z*_* = 0.35), and moral justification (*p* < 0.001, Cohen’s *d_*z*_* = 0.30; *p* < 0.001, Cohen’s *d_*z*_* = 0.31). Finally, moral justification scored higher than dehumanization (*p* < 0.001, Cohen’s *d_*z*_* = 0.16).

#### Mean Victim

The third ANOVA showed a significant main effect. Bonferroni *post hoc* procedures revealed that victim blaming was significantly higher than dehumanization (*p* < 0.001, Cohen’s *d_*z*_* = 0.99), diffusion of responsibility (*p* < 0.001, Cohen’s *d_*z*_* = 0.75), euphemistic labeling (*p* < 0.001, Cohen’s *d_*z*_* = 0.74), distorting consequences (*p* < 0.001, Cohen’s *d_*z*_* = 0.72), advantageous comparison (*p* < 0.001, Cohen’s *d_*z*_* = 0.57), moral justification (*p* < 0.001, Cohen’s *d_*z*_* = 0.56), and displacement of responsibility (*p* < 0.001, Cohen’s *d_*z*_* = 0.52).

Furthermore, moral justification was significantly higher than dehumanization (*p* < 0.001, Cohen’s *d_*z*_* = 0.72), diffusion of responsibility (*p* < 0.001, Cohen’s *d_*z*_* = 0.43), euphemistic labeling (*p* < 0.001, Cohen’s *d_*z*_* = 0.39), distorting consequences (*p* < 0.001, Cohen’s *d_*z*_* = 0.34), displacement of responsibility (*p* = 0.002, Cohen’s *d_*z*_* = 0.21), and advantageous comparison (*p* = 0.002, Cohen’s *d_*z*_* = 0.15). Advantageous comparison scored higher than dehumanization (*p* < 0.001, Cohen’s *d_*z*_* = 0.59), diffusion of responsibility (*p* < 0.001, Cohen’s *d_*z*_* = 0.36), euphemistic labeling (*p* < 0.001, Cohen’s *d_*z*_* = 0.31), distorting consequences (*p* < 0.001, Cohen’s *d_*z*_* = 0.23), and displacement of responsibility (*p* < 0.001, Cohen’s *d_*z*_* = 0.10). Displacement of responsibility and distorting consequences scored higher than dehumanization (*p* < 0.001, Cohen’s *d_*z*_* = 0.41; *p* < 0.001, Cohen’s *d_*z*_* = 0.41), diffusion of responsibility (*p* < 0.001, Cohen’s *d_*z*_* = 0.21; *p* < 0.001, Cohen’s *d_*z*_* = 0.20), and euphemistic labeling (*p* = 0.031, Cohen’s *d_*z*_* = 0.12; *p* = 0.029, Cohen’s *d_*z*_* = 0.12). Finally, diffusion of responsibility scored higher than dehumanization (*p* < 0.001, Cohen’s *d_*z*_* = 0.28).

#### Likable Victim

The fourth ANOVA showed a significant main effect. *Post hoc* tests using the Bonferroni correction revealed that euphemistic labeling was higher than dehumanization (*p* < 0.001, Cohen’s *d_*z*_* = 0.58), victim blaming (*p* < 0.001, Cohen’s *d_*z*_* = 0.57), moral justification (*p* < 0.001, Cohen’s *d_*z*_* = 0.55), diffusion of responsibility (*p* < 0.001, Cohen’s *d_*z*_* = 0.41), distorting consequences (*p* < 0.001, Cohen’s *d_*z*_* = 0.31), and advantageous comparison (*p* < 0.001, Cohen’s *d_*z*_* = 0.20).

Displacement of responsibility, advantageous comparison, and distorting consequences were higher than dehumanization (*p* < 0.001, Cohen’s *d_*z*_* = 0.57; *p* < 0.001, Cohen’s *d_*z*_* = 0.52; *p* < 0.001, Cohen’s *d_*z*_* = 0.48), victim blaming (*p* < 0.001, Cohen’s *d_*z*_* = 0.50; *p* < 0.001, Cohen’s *d_*z*_* = 0.49; *p* < 0.001, Cohen’s *d_*z*_* = 0.46), moral justification (*p* < 0.001, Cohen’s *d_*z*_* = 0.42; *p* < 0.001, Cohen’s *d_*z*_* = 0.43; *p* < 0.001, Cohen’s *d_*z*_* = 0.41), and diffusion of responsibility (*p* < 0.001, Cohen’s *d_*z*_* = 0.32; *p* < 0.001, Cohen’s *d_*z*_* = 0.27; *p* < 0.001, Cohen’s *d_*z*_* = 0.24). Diffusion of responsibility was higher than dehumanization (*p* < 0.001, Cohen’s *d_*z*_* = 0.47), victim blaming (*p* < 0.001, Cohen’s *d_*z*_* = 0.34), and moral justification (*p* < 0.001, Cohen’s *d_*z*_* = 0.19). Finally, moral justification scored higher than dehumanization (*p* < 0.001, Cohen’s *d_*z*_* = 0.18).

## Discussion

Social–cognitive theory (Bandura, 2016) adopts an interactionist perspective on morality, assuming an interplay between personal, behavioral, and environmental influences. Although moral disengagement tends to develop into trait-like habitual patterns (Bandura, 1999, 2016), its mechanisms should not be considered fixed, stable, or static personality traits because they can change over time (Thornberg et al., in press) and—as the theory argues (Bandura, 2016)—they can also be activated differently across various situations due to different social cues or conditions (Bandura, 2016). To our knowledge, the current study is the first to examine whether the levels of endorsement of different moral disengagement mechanisms vary across different conditions of verbal bullying by adopting a repeated within-subjects experimental design and a vignette technique.

### Moral Justification, Victim Blaming, and Dehumanization

As anticipated, the students were more inclined to morally justify their bullying behavior in the hypothetical condition in which they perceived that the victim had been mean to their friend than in the other three bullying conditions. A possible explanation might be that this “mean victim” cue functions like an aggression cue (Huesmann, 2018) that, together with a perception of facing injustice and moral violation (Hoffman, 2000; Nucci, 2001), increases the probability of reactions like empathic anger, feelings of injustice, and moral outrage (Hoffman, 2000; Batson et al., 2007; Pozzoli et al., 2017). Moral justification then takes the form of perceiving bullying perpetration toward the mean victim as serving a moral purpose (retributive justice) and cognitively restructuring this behavior as punitive aggression, upstanding, or revenge rather than bullying (cf., Bandura, 1999, 2016). As expected, students were more inclined to blame the victim (who then “deserves” his or her suffering) in the mean victim condition than in the other three bullying conditions. Victim blaming seems to go hand in hand with moral justification and, in the mean victim condition, these were the two most salient mechanisms of moral disengagement, which supported our expectation. Victim blaming scored highest, followed by moral justification as the second highest mechanism.

Aggression and externalizing behaviors have been found to predict peer victimization among children and adolescents (Frey and Strong, 2018; Pouwels et al., 2019). These links may be mediated, at least in part, by victim blaming and moral justification because these children risk being perceived by other children as “mean” peers, who “deserve” to be repeatedly victimized and therefore have only themselves to blame for being targets of “justified” punitive aggression. Further research should examine how moral justification and victim blaming in particular are associated with bullying directed toward so-called “aggressive victims.” In addition, previous research has found that moral justification (Robson and Witenberg, 2013; Thornberg and Jungert, 2014; Bjärehed et al., 2020), dehumanization, and victim blaming are associated with bullying behavior (Thornberg and Jungert, 2014; Bjärehed et al., 2020). Therefore, those who bully others might be psychologically motivated to perceive their victims as “mean” and “guilty.” Thus, in order to cognitively restructure their bullying perpetration as standing up for themselves and/or others (moral justification), there might be a need to cognitively restructure the victim as “mean” and therefore someone who deserves to be punished (victim blaming).

Moreover, as hypothesized, dehumanization scored higher in the mean victim condition than in the other three conditions, indicating that it seems easier for students to dehumanize victims of bullying when they are perceived as aggressive or mean. Victim blaming and the perception that the victim is mean could even result in the more extreme form of *demonization*, in which the victim is condemned as evil and wicked—a perception that fuels the belief in and endorsement of redemptive violence against the evil victim, who fully deserves the inhumane treatment and harsh punishment (Li et al., 2014; Webster and Saucier, 2015). Further research should explore how dehumanization, and its more extreme form of demonization, might be involved in bullying and might interact with victim blaming and moral justification, particularly when the targets are aggressive victims or members of stigmatized social categories.

We expected that the students would be less inclined to use dehumanization and victim blaming in the likable victim condition because it should be difficult to dehumanize and blame someone whom they actually like and perceive as being kind to everyone. The current findings confirmed this hypothesis by showing that both dehumanization and victim blaming scored lower in the likable victim condition than in the other three conditions. In addition, dehumanization, victim blaming and moral justification scored lower than the other moral disengagement mechanisms in the likable victim condition (and dehumanization scored lower than moral justification). These findings suggest that students high in likability or sociometric popularity (well-liked by their peers and high in prosocial behavior; see Closson and Hymel, 2016; van den Broek et al., 2016) are at lower risk of being targets of dehumanization and victim blaming from their peers, and it seems more difficult for students to morally justify bullying directed toward a well-liked and friendly peer. These findings can be compared with studies showing that students who are more well-liked and prosocial are less likely to be peer victimized (de Bruyn et al., 2010; van der Ploeg et al., 2015; Pouwels et al., 2016; Babarro et al., 2017; Ma et al., 2020).

### Euphemistic Labeling

In accordance with our hypothesis, the present findings show that euphemistic labeling scored higher in the laughing audience condition and the likable victim condition than in the other two bullying conditions. As expected, euphemistic labeling and displacement of responsibility scored higher than all the other mechanisms of moral disengagement in the laughing audience condition. In other words, the students seemed to be more inclined to label their bullying behavior as “just kidding” and “joking” when bystanders were present and acted as reinforcers (watching and laughing). This result suggests that the presence of reinforcing bystanders (Salmivalli, 2010) contributes to trivializing and normalizing bullying as non-serious or playful jokes (Thornberg and Delby, 2019). The laughing audience communicates to the bully that he or she is “just kidding” and “joking” and thus helps the bully to cognitively restructure his or her bullying behavior as fun entertainment—a euphemistic labeling that blurs the boundary between joke and abuse.

The expectation that euphemistic labeling would score higher than all the other mechanisms of moral disengagement in the likable victim condition was also supported. We assumed at the outset of the current study that bullying a person whom students really like and who is friendly and kind to others would in general make it more difficult for moral disengagement to be activated to convince them that the behavior is acceptable. In line with that assumption, our initial results revealed that moral disengagement as a global construct was lower in the likable victim condition than in the other three bullying conditions. However, one way to avoid moral self-sanctions (Bandura, 1999, 2016) would be to cognitively restructure the bullying behavior as “friendly teasing” and “just joking” (cf., Billig, 2005). Such euphemistic labeling can help bullies to reduce their cognitive dissonance (Festinger, 1962; McGrath, 2017) and its unwanted negative emotions, including moral distress (Brüggemann et al., 2019; Gini et al., 2020) and feelings of guilt, remorse, and self-condemnation (Bandura, 1999, 2016), by convincing themselves that they are not being mean and aggressive toward their peer at all but are “just kidding.”

### Displacement of Responsibility

We hypothesized that displacement of responsibility and diffusion of responsibility would score higher than the other moral disengagement mechanisms in the group conformity condition. We found this to be true for the displacement of responsibility. The current results also reveal that displacement of responsibility, together with euphemistic labeling, scored higher than the other moral disengagement mechanisms in the laughing audience condition. Furthermore, students were more prone to displace responsibility to adults in the group conformity and laughing conditions than in the likable victim condition, but there were no significant differences in relation to the mean victim condition, which partly supports our expectation. Both group conformity and laughing audience conditions include the presence of peers who express pro-bullying norms (Saarento and Salmivalli, 2015). A group of bullies and a laughing audience not only mediate social approval but also make the actual bullying more visible and significant in the school setting. When there is a whole group of students engaged in bullying perpetration, or when those who are bullying are supported and cheered on by other students, it would seem reasonable to assume that an individual bully would be more inclined to interpret his or her bullying behavior as socially acceptable in school and that teachers and other school staff are therefore responsible for allowing it to happen. If such vivid, visible, and noticeable bullying can continue, students might perceive it as an instance of poor rule enforcement in which teachers and other school staff allow bullying to occur (Cunningham et al., 2016; Thornberg and Delby, 2019). This can be understood as the emergence of a “culture of bullying” that supports or encourages bullying behavior because students believe that “in most cases, neither their classmates nor their teachers would intervene to stop bullying” (Unnever and Cornell, 2003, p. 18). Thus, not only bystanders but also bullies might engage in *responsibility transfer* to teachers; in other words, they feel that teachers are responsible for the bullying and for stopping it (cf., Bellmore et al., 2012).

### Diffusion of Responsibility

In accordance with our hypothesis, diffusion of responsibility scored higher in the group conformity condition than in the other three bullying conditions. The expectation that both displacement of responsibility and diffusion of responsibility would score higher than the other mechanisms in the group conformity condition was only partly confirmed: displacement of responsibility scored highest, while diffusion of responsibility, together with euphemistic labeling and advantageous comparison, scored higher than the other four mechanisms. The fact that diffusion of responsibility was higher in the group conformity condition than in the other three bullying conditions indicates that this mechanism is probably more involved when bullying is conducted by a group than by an individual. Olweus (1993) refers to diffusion of responsibility as a possible explanation for why otherwise nice and non-aggressive students conform to the group behavior of bullying: “It is well-known from social psychology that a person’s sense of individual responsibility for a negative action such as bullying may be considerably reduced when several people participate” (p. 44). When an inhumane act is committed by a group, the group nature of the action provides a sense of anonymity and diffuses, and thus diminishes, any sense of personal responsibility (Bandura, 2016). Our findings suggest that bullies are more inclined to experience diffusion of responsibility when many people are acting together in bullying.

### Advantageous Comparison and Distorting Consequences

We did not have any particular hypothesis regarding advantageous comparison or distorting consequences in the present study. However, our findings revealed that both mechanisms scored higher in the mean victim condition than in the likable victim, laughing audience, and group conformity conditions. Even though victim blaming and moral justification scored higher than advantageous comparison, advantageous comparison scored higher than the other mechanisms in the mean victim condition. Together with displacement of responsibility, distorting consequences scored higher than dehumanization, diffusion of responsibility, and euphemistic labeling in the same condition. Altogether, advantageous comparison and distorting consequences seem to be triggered or activated when the victim is perceived as someone who has been mean, even though there are other mechanisms that are activated to a greater degree. A possible explanation might be that victim blaming and moral justification are more salient in this bullying condition, indicating that bullying might be perceived by the actor as a moral action. The presence of these two mechanisms makes it possible for the actor to make advantageous comparisons: bullying a mean victim who “deserves” to be punished and, through this action, avenging and standing up for a friend is acceptable (or even a good thing to do) as compared to bullying an innocent victim.

A possible explanation for why distorting consequences was higher in the mean victim condition than in the other three conditions could be that children and adolescents may be less inclined to empathize or sympathize with someone whom they perceive to be mean and having only him- or herself to blame (victim blaming tends to reduce feelings of empathy; see Hoffman, 2000), and they might therefore be more prone to ignore, minimize, or disregard the harmful effects of their actions toward that person. They simply do not care about the mean victim’s suffering. In addition, considering that dehumanization scored higher in the mean victim condition than in the other three conditions, this mechanism might also contribute to distorting the consequences. Dehumanization is associated with diminished empathy for those who are dehumanized (Haslam and Loughnan, 2014), which in turn facilitates minimizing, distorting, or disregarding the harm and suffering that one causes the victim.

### Study Limitations

Some limitations of this study should be noted. Firstly, the variables have been assessed through self-reporting, which is vulnerable to social desirability and intentionally exaggerated responses (Cornell and Bandyopadhyay, 2010), even though the questionnaire was anonymous. However, so far in the literature, there are not alternative valid and reliable manners to assess students’ endorsement of moral disengagement mechanisms. Secondly, it is important to recognize that examining how adolescents respond to hypothetical scenarios is not the same as investigating how they would respond in real-life situations. The ecological validity is therefore somewhat threatened (Cicourel, 1982). However, the vignette technique is widely used in the social and educational psychology literature and in the field of bullying and peer aggression research in particular because it enables researchers to collect responses to the same situations from all of the participating children and to manipulate such situations experimentally (which is impossible or unethical to do in real life). In addition, some studies have demonstrated that how children and adolescents respond in real-life situations generally corresponds with how they respond in hypothetical situations (Smetana et al., 1993; Turiel, 2008), a result that addresses the ecological validity concerns. Moreover, asking questions about hypothetical events instead of real-life situations in which children and adolescents themselves have been involved as participants should be less intrusive and reduce the risk of social desirability bias, considering this study’s sensitive topic of bullying. Another limitation concerning the experimental material deals with the fact that, for sake of simplicity, we limited the scenarios to instances of verbal bullying. Because it has been found that students’ judgments of bullying can vary across different forms of bullying (e.g., Gini et al., 2008), future studies should try to confirm and expand the current findings with other forms of bullying behavior. Finally, a note of caution needs to be sounded regarding the generalization of the findings. There was a participation rate of 42%. In particular, more girls than boys participated in the study, although the original sample contained more boys, which indicates a selection bias. Despite that, the final sample represented a span of students aged 10–20 years with different socioeconomic (ranging from lower to upper-middle class) and socio-geographic backgrounds. Still, this sample of children and adolescence from certain areas of Sweden may or may not be similar to the population of children and adolescents whom readers primarily work with or are interested in studying.

### Implications for Practice

Most bullying episodes happen is schools, and highlighting the mechanisms associated with different manifestations of the phenomenon may help informing prevention programs more focused on morality. On one hand, it would be important to address distortions in morality with the aims of favoring adolescents’ moral engagement and of promoting the understanding of personal responsibility. For example, this could be achieved by regularly engaging students in discussions about moral issues, about what it means to be a caring, fair, and responsible person (Oser, 1986). Moreover, another strategy that can be implemented within the class is the use of curricular activities during regular lessons to raise students’ awareness about bullying behavior and about their personal role in the group dynamics. These lessons should also aim at changing students’ understanding of the victim’s perspective (Kärnä et al., 2011). On the other hand, the present results suggest that anti-bullying prevention program could also benefit from more tailored intervention strategies that adopt a “who-bullies-whom” approach (Rodkin and Berger, 2008; Tolsma et al., 2013) by taking into account the specific characteristics of the students directly involved (e.g., whether the victimized student is liked or rejected, or whether he or she is aggressive toward other peers or just “passive”). This approach could, for example, entail specific strategies for individual students or small groups, such as assertiveness training and social skills training (e.g., through role-play exercises).

## Conclusion

Despite these limitations, this study extends the knowledge of moral disengagement and its mechanisms in relation to bullying among children and adolescents. To our knowledge, it is the first to examine whether the levels of different moral disengagement mechanisms vary across different conditions of bullying. Our results demonstrate changes in moral disengagement mechanisms *across* the bullying conditions as well as differences between moral disengagement mechanisms *within* each bullying condition. In accordance with the main assumptions of social–cognitive theory (Bandura, 1999, 2016), the current findings provide evidence that moral disengagement mechanisms can be selectively activated under different circumstances. This, in turn, supports the assumption that moral agency and moral disengagement are produced by the reciprocal interplay between individual and situational factors.

## Data Availability Statement

The datasets generated for this study are available on request to the corresponding author.

## Ethics Statement

The studies involving human participants were reviewed and approved by Regional Ethical Review Board at Linköping. Written informed consent to participate in this study was provided by the participants’ legal guardians.

## Author Contributions

All authors listed have made a substantial, direct and intellectual contribution to the work, and approved it for publication.

## Conflict of Interest

The authors declare that the research was conducted in the absence of any commercial or financial relationships that could be construed as a potential conflict of interest.

## Footnotes

1 Originally, there were six vignettes but two were excluded to simplify the design and to lower the risk of multiplicity or mass significance effect.

## Appendix: Questionnaire A

### Pretend That You Are a Person Who Is Teasing Others

Here come four short stories that we want you to identify yourself with. We want you to pretend to be a person who teases others and who is more popular, powerful and stronger than the person you are teasing. Don’t worry about whether you have done any of these things before or not. Just imagine in each story that it is you who is doing it.

Imagine that your friends are teasing another student at school. When you are with your friends, you tease this student as well a couple of times a week.

Imagine that you are teasing another student a couple of times a week. Those who are watching are laughing and think you are fun.

Imagine a student who talks badly about your friend. You begin to tease that student a couple of times a week.

Imagine a person whom you like and who is nice and kind to all your classmates. You begin to tease that student a couple of times a week.

In the questionnaire, each vignette was followed by the following question, “How would you think and feel then?”, and the same moral disengagement scale:

**Table:**

	disagree agree
1. Um, I was just kidding with him/her.	➀ ➁ ➂ ➃ ➄ ➅ ➆
2. Well, it’s not my fault because a lot of others are doing it to him/her too.	➀ ➁ ➂ ➃ ➄ ➅ ➆
3. Well, the kid has him-/herself to blame.	➀ ➁ ➂ ➃ ➄ ➅ ➆
4. It’s no big deal. Nobody gets hurt.	➀ ➁ ➂ ➃ ➄ ➅ ➆
5. I wouldn’t care because he/she is not like the rest of us.	➀ ➁ ➂ ➃ ➄ ➅ ➆
6. It feels okay because there are so much worse things that you could do to a person.	➀ ➁ ➂ ➃ ➄ ➅ ➆
7. Well, it’s actually the teachers’ fault because they are not there and watching what we are doing.	➀ ➁ ➂ ➃ ➄ ➅ ➆
8. Feels good. I’m doing it for a good cause.	➀ ➁ ➂ ➃ ➄ ➅ ➆
9. I’m doing a good thing because I do it for a good reason.	➀ ➁ ➂ ➃ ➄ ➅ ➆
10. Well, it’s the adults’ fault. They do nothing about it.	➀ ➁ ➂ ➃ ➄ ➅ ➆
11. Well, it’s not that bad if you compare it to hitting and kicking a person.	➀ ➁ ➂ ➃ ➄ ➅ ➆
12. It’s okay because he/she is worth less than the rest of us.	➀ ➁ ➂ ➃ ➄ ➅ ➆
13. Well, he/she wouldn’t really get sad.	➀ ➁ ➂ ➃ ➄ ➅ ➆
14. It’s actually his/her own fault.	➀ ➁ ➂ ➃ ➄ ➅ ➆
15. Because other classmates are teasing the person, I can’t be blamed for doing that too.	➀ ➁ ➂ ➃ ➄ ➅ ➆
16. It’s okay because it’s just a joke.	➀ ➁ ➂ ➃ ➄ ➅ ➆
